# Employment Status, Self-Rated Health, and Stress Among Family Caregivers of Community-Dwelling Care Recipients in Japan

**DOI:** 10.3390/healthcare14183036

**Published:** 2026-09-16

**Authors:** Kanna Katsumata, Junko Hoshino, Mika Sakurai

**Affiliations:** 1 Department of Integrated Health Sciences, Nagoya University Graduate School of Medicine, Nagoya 461-8673, Japan; katsumata.kanna.c5@s.mail.nagoya-u.ac.jp (K.K.); msakurai87@gifu-cn.ac.jp (M.S.); 2 Department of Nursing, Gifu College of Nursing, Gifu 501-6295, Japan

**Keywords:** family caregiver, employment, self-rated health, stress

## Abstract

**Background/Objectives**: With population aging, increasing attention has been directed toward family caregivers balancing paid employment and caregiving responsibilities. This study aimed to investigate the association between employment status and self-rated health and stress among family caregivers and to explore associations between caregiving-related factors and these outcomes according to employment status. **Methods**: This study included 409 family caregivers of patients receiving home-visit nursing services in Japan. Data on caregiver characteristics, self-rated health, stress during the past month, and caregiving-related factors, including caregiving situations, support situations, and negative appraisal of caregiving assessed using the Negative Appraisal of Care (NAC) Scale, were collected. Logistic regression analyses with inverse probability weighting were conducted to examine the association between employment status and health outcomes. Exploratory stratified univariate analyses were performed by employment status to examine associations between caregiving-related factors and self-rated health and stress. **Results**: Of the 409 participants, 126 were working carers and 283 were non-working carers. After IPW and multivariable adjustment, employment status was not significantly associated with self-rated health, whereas working carers were significantly more likely to report stress than non-working carers. In the exploratory stratified analyses, among working carers, poor self-rated health was more frequently observed among those without additional caregivers, those using short-stay services, and those with higher NAC scores. Stress was also more frequently observed among those without additional caregivers. Among non-working carers, poor self-rated health was more frequently observed among those with higher NAC scores, whereas stress was more frequently observed among female caregivers, those caring for recipients with cognitive impairment, and those using short-stay services. **Conclusions**: Working carers reported higher levels of stress than those of non-working carers, even after adjustment for caregiving-related factors. Exploratory analyses among working carers suggested that the presence of additional caregivers was associated with both self-rated health and stress, indicating that caregiving support may be an important factor in balancing employment and caregiving responsibilities.

## 1. Introduction

According to the United Nations World Population Prospects 2024, the global population aged 65 years and older exceeded 800 million as of July 2023 and is projected to double to approximately 1.6 billion by 2050 [1]. With population aging, the demand for care is increasing worldwide. The World Health Organization (WHO) has pointed out that current care provision relies heavily on informal care, which is primarily provided by family members, particularly women [2]. Family caregivers often receive insufficient support and experience substantial physical and psychological burdens [3], and the health problems of caregivers themselves have become a global issue. Common health problems reported among family caregivers include increased psychological stress and depressive symptoms as well as poorer subjective well-being and physical health [4].

In response to these challenges, the United Nations and WHO designated the period from 2021 to 2030 as the UN Decade of Healthy Ageing, emphasizing the importance of comprehensive support for family caregivers and communities [5]. This initiative includes the expansion of formal care services and the promotion of flexible working arrangements to enable older adults to continue living independently in their familiar environments [6].

Within this global context, Japan is experiencing population aging at an unprecedented rate compared with other countries. As of September 2025, the proportion of older adults in the country has reached 29.4% [7]. In addition, both the number of individuals requiring long-term care and the number of family caregivers continue to increase [8,9]. According to the 2022 Employment Status Survey, approximately 6.29 million people in Japan were engaged in family caregiving [10]. Meanwhile, although the population aged 65 years and older is expected to peak in the future, the working-age population (aged 15–64 years) is projected to decline rapidly [11], making securing caregiving resources an increasingly critical issue.

Against this societal backdrop, in recent years, increasing attention has been paid to “working carers,” individuals who balance paid employment with caregiving responsibilities. According to the *Working Carers Guide* published by Carers Wales, a working carer is defined as “anyone who provides care and support for another person or persons while doing paid work” [12]. The number of working carers in Japan is also expected to increase. The Ministry of Economy, Trade and Industry estimates that by 2030, approximately 3.18 million out of the 8.33 million family caregivers will be working carers. Furthermore, difficulties in balancing work and caregiving are projected to result in economic losses exceeding JPY 9 trillion by 2030 owing to reduced labor productivity [13]. These findings highlight that the challenges associated with balancing work and caregiving are no longer solely individual issues but should be addressed as a societal concern.

However, the concept of “working carers” is relatively new, and academic research focusing specifically on their health status remains limited. Previous studies have reported that working carers have higher scores on health-related quality of life measures such as the SF-36v2 Health Survey, than non-working carers, suggesting a better subjective health status [14]. Conversely, other studies have shown that working carers exhibit higher scores on the Hospital Anxiety and Depression Scale, indicating greater levels of anxiety and depression than non-working carers [15].

Despite these findings, differences in health status according to employment status among caregivers and the associations between caregiving-related factors and health outcomes according to employment status have not yet been sufficiently clarified.

These inconsistent findings may be considered from theoretical perspectives on the effects of occupying multiple roles. Goode introduced the concept of role strain, which refers to difficulties in meeting the expectations and demands associated with multiple social roles [16]. In particular, competing role demands may lead to role conflict and increased psychological burden. Alternatively, role expansion theory suggests that occupying multiple roles may generate additional resources that can support the fulfillment of diverse demands [17]. Employment may provide resources, such as income, social interaction, and opportunities outside the caregiving role, which may have beneficial effects on caregivers’ health and well-being. These contrasting theoretical perspectives provide a possible framework for understanding the inconsistent findings regarding the association between employment status and caregiver health. Based on role strain theory, we hypothesized that working carers would be more likely than non-working carers to report poor self-rated health and stress during the past month.

Japan has the highest aging rate in the world and has developed support systems based on long-term care insurance and community-based integrated care. However, research findings on the health of working carers in Japan have not been sufficiently disseminated internationally. Therefore, this study aimed to examine the association between employment status and self-rated health and stress among family caregivers of patients receiving home-visit nursing services. In addition, this study explored associations between caregiving-related factors and health outcomes according to employment status.

## 2. Materials and Methods

This study used a portion of the data from a cross-sectional study conducted in Japan between January 2022 and January 2025, titled “Experiences of Family Caregivers of People with Formal Long-term Care Needs Receiving Home-visit Nursing [18,19].”

### 2.1. Participants

The study participants were family caregivers who provided care to patients receiving home-visit nursing services in selected prefectures across Japan. Family caregivers aged 20 years or older who were able to understand the study information and provided informed consent to participate were included in this study.

Of the 418 responses collected, nine participants with missing employment status data were excluded, resulting in a final analytic sample of 409 participants.

### 2.2. Data Collection

Data were collected using an anonymous self-administered questionnaire. First, Japan was divided into eight geographic regions, and 34 of the 47 prefectures in Japan were randomly selected from these regions. Home-visit nursing stations located in these prefectures were identified as potential study sites. Overall, 373 home-visit nursing stations were randomly selected using the “Nursing Care Service Information Search System” provided by the Ministry of Health, Labour and Welfare [20]. The administrators of the selected facilities were contacted via telephone to explain the study and to confirm whether they would accept the study cooperation request documents. Study cooperation consent forms were sent to 239 facilities that agreed to participate. Agreement to participate was confirmed upon receipt of the signed consent form. Ultimately, 84 facilities agreed to participate, and 2254 questionnaires and study information sheets were distributed to potential participants. The questionnaires were distributed to caregivers via visiting nurses. The participants completed the questionnaires at home and returned them directly to the researchers via mail. In total, 418 questionnaires were returned.

### 2.3. Measures

The questionnaire was administered in Japanese and included items related to caregiver characteristics, caregiver health, employment status, work environment, caregiving situations, support situations, and caregiver burden.

#### 2.3.1. Caregiver Characteristics

Variables included the caregiver’s age, gender, relationship with the care recipient (spouse/non-spouse), and the presence of cohabiting family members.

#### 2.3.2. Caregiver Health

Health indicators included self-rated health and perceived stress over the past month. Self-rated health was assessed using the question, “How would you rate your current health status?” with four response options (“very good,” “good,” “not very good,” and “poor”). Those who responded as “very good” and “good” were categorized as “healthy,” while those who answered as “not very good” and “poor” were categorized as “unhealthy.” Perceived stress over the past month was assessed using the question, “Have you experienced stress in the past month?” with four response options (“very much,” “somewhat,” “not much,” and “not at all”). Those who responded as “very much” and “somewhat” were categorized as “stressed,” while those who answered as “not much” and “not at all” were categorized as “not stressed.”

#### 2.3.3. Employment Status

Employment status was classified based on the responses to employment-related questions. Participants who reported being “self-employed,” “regularly employed,” or “non-regularly employed (part-time or temporary)” were categorized as “employed (working carers),” whereas those who reported being “unemployed” were categorized as “not employed (non-working carers).”

#### 2.3.4. Work Environment

The work environment of working carers was assessed based on their ability to adjust working hours for caregiving. Participants were asked, “Are you able to adjust your working hours to provide care?” Responses were recorded on a 4-point scale. Those who answered “able” were classified as “able to adjust,” while those who answered “somewhat able,” “not very able,” or “not able at all” were classified as “unable to adjust.”

#### 2.3.5. Caregiving Situations

Variables included care recipient age, gender, level of care need (support level 1 to care level 2/care level 3 or higher) [21], duration of caregiving (months), daily caregiving time (half-day to full-day/≤2–3 h), and presence of cognitive impairment in the care recipient.

#### 2.3.6. Support Situations

Support-related variables included the presence of additional caregivers, use of home-based services (home-visit rehabilitation, home-visit care, and home-visit bathing), use of daycare services (day care and day rehabilitation), and use of short-stay services. The presence of additional caregivers was assessed using the question “Is there anyone other than yourself who provides care?” with response options of “yes” or “no.” The participants were also asked about the frequency of their use of home-based and daycare services per week and their use of short-stay services within the past year.

#### 2.3.7. Caregiver Burden

Caregiver burden was assessed using the Negative Appraisal of Care (NAC) Scale developed by Yamamoto et al. [22]. The NAC Scale was originally developed and validated in Japan for use with Japanese family caregivers. This scale measures caregivers’ perceptions of the difficulties and distress associated with caregiving and consists of four domains comprising 14 items: “role exhaustion,” “social isolation,” “difficulty coping with symptoms,” and “relationship with the care recipient.” Each item was rated on a 4-point scale ranging from 0 (“not at all applicable”) to 3 (“very applicable”), and the domain scores were converted to scores on the 0–100 scale. In this study, the total NAC score (0–100) was calculated as the average of the four domain scores. Higher scores indicate a greater negative appraisal of caregiving. The reliability coefficients of the NAC Scale were 0.87 in the original study [22] and 0.920 in a subsequent study of Japanese family caregivers [19]. In the current study, the Cronbach’s alpha coefficient for the 14-item NAC Scale was 0.894.

### 2.4. Statistical Analysis

Descriptive statistics were calculated for caregiver characteristics, caregiving situations, and health indicators. Comparisons between the employed (working carers) and unemployed (non-working carers) groups were conducted as follows. For continuous variables, normality was assessed using the Shapiro–Wilk test, and either an independent samples *t*-test or the Mann–Whitney *U* test was applied. For categorical variables, the chi-square test was used, and Fisher’s exact test was applied when the expected cell counts were less than five.

To examine the association between employment status and health, inverse probability weighting (IPW) based on propensity scores was applied. Previous studies have suggested that working carers and non-working carers may differ in background characteristics, particularly caregiver age [14,15]; therefore, propensity scores were used to adjust for background characteristics. Propensity scores (PS) were estimated using logistic regression analysis, with employment status as the dependent variable and caregiver age, relationship with the care recipient, and presence of cohabiting family members as independent variables. Employment status (working vs. non-working) was treated as the exposure. The independent variables were selected based on Katz’s recommendation to include variables associated with exposure assignment in the propensity score model [23] and on previous studies reporting associations between these characteristics and employment status [15,24]. Caregiver age, relationship with the care recipient, and presence of cohabiting family members were treated as pre-exposure confounders—background characteristics present before employment status was determined—and were entered into the propensity score model. The average treatment effect (ATE) in the study population was targeted using unstabilized inverse probability weights, calculated as 1/PS for working carers and 1/(1 − PS) for non-working carers. The distributions of propensity scores and IPW weights were examined for extreme values. Overlap of the propensity score distributions between working and non-working carers was visually assessed using histograms to evaluate the positivity assumption. The discrimination of the propensity score model was evaluated using the area under the receiver operating characteristic curve (AUC). Covariate balance before and after IPW was assessed using standardized mean differences (SMDs). The effective sample size after weighting was also calculated to assess the influence of variability in the weights.

After applying IPW, logistic regression analyses were conducted to examine the association between employment status and health. Self-rated health and stress over the past month were analyzed as dependent variables. In the univariate analyses, employment status was included as the independent variable. In the multivariable analyses, care recipient age, level of care need, and duration of caregiving were additionally included as covariates based on previous studies [25,26]. In addition, exploratory stratified analyses were conducted separately for working and non-working carers to examine associations between selected variables and health indicators. The variables included in these analyses were selected based on previous studies [14,15,24,25,26,27,28,29,30,31,32,33,34,35,36,37,38,39,40,41,42]. With regard to self-rated health, the variables examined included caregiver characteristics, work environment, caregiving situations, support situations, and caregiver burden. Regarding stress over the past month, the variables examined included caregiver characteristics, work environment, caregiving situations, and support situations. These stratified analyses consisted of separate univariate comparisons within each group. The work environment (ability to adjust working hours for caregiving) was analyzed only among working carers.

Participants with missing or invalid values for variables required in a specific analysis were excluded from that analysis, and no imputation of missing data was performed. All statistical analyses were performed using IBM SPSS Statistics version 30, and statistical significance was set at *p* < 0.05.

### 2.5. Ethical Considerations

Study participation was voluntary. The participants were informed that they could refuse to participate or withdraw from the study at any time without any disadvantages. The study was approved by the Research Ethics Committee of Nagoya University. Ethical approval was obtained both for the original research project (Approval No. 21-138-2, date of approval: 30 March 2022) and for the present study (Approval No. 25-101, date of approval: 9 June 2025).

## 3. Results

### 3.1. Participant Characteristics

Table 1 presents the characteristics of the 409 caregivers included in the analysis. The mean age (±standard deviation) of the participants was 68.4 ± 11.8 years, and 72.9% were female. Regarding the caregivers’ relationship with the care recipient, 51.5% were spouses, and 74.1% lived with the care recipient. In terms of caregiver health, 69.4% of the participants were classified as healthy. Meanwhile, 80.7% of the participants reported experiencing stress in the past month.

Table 2 shows the characteristics of the working and non-working carers. Of the 409 participants, 126 (30.8%) were classified as working carers and 283 (69.2%) as non-working carers.

Regarding basic characteristics, the mean age of working carers was 60.9 ± 10.6 years, which was significantly lower than that of non-working carers (71.8 ± 10.8 years, *p* < 0.001). In addition, the proportion of non-spousal caregivers was higher among working carers than among non-working carers (*p* < 0.001).

Regarding health indicators, the proportion of participants classified as healthy was higher in the working carer group (*p* = 0.004). The proportion of participants who reported stress in the past month was also higher in the working carer group (*p* = 0.008).

### 3.2. Evaluation of the Propensity Score Model

The AUC of the propensity score model was 0.784, indicating acceptable-to-good discriminative ability [43]. Propensity scores ranged from 0.06 to 0.91 among working carers and from 0.03 to 0.92 among non-working carers. Visual inspection of the histograms showed overlap in the propensity score distributions between the two groups, supporting the positivity assumption. No observations were trimmed based on propensity scores. The IPW weights ranged from 1.03 to 15.72, with a mean of 1.98 (SD = 1.73), and no truncation of the weights was performed. The effective sample size after weighting was 220.5.

### 3.3. Balance Assessment of Covariates

Table 3 presents the SMDs before and after the IPW. Prior to weighting, several covariates exceeded the commonly accepted threshold for good balance (SMD < 0.10) [44,45]. After applying IPW, the SMDs for caregiver age, relationship with the care recipient, and presence of cohabiting family members were all below 0.10, whereas the SMD for caregiver gender was 0.13. Overall, covariate balance improved after weighting.

### 3.4. Association Between Employment Status and Self-Rated Health and Related Factors

Table 4 presents the association between employment status and self-rated health after applying IPW. In the IPW-adjusted logistic regression analysis, employment status was significantly associated with self-rated health (odds ratio [OR] = 1.90, 95% confidence interval [CI]: 1.37–2.63, *p* < 0.001). However, in the multivariable analysis with adjustments for care recipient age, level of care need, and duration of caregiving, the association was not statistically significant (OR = 1.41, 95% CI: 0.98–2.03, *p* = 0.066).

Table 5 presents the results of the exploratory univariate analyses of self-rated health stratified by employment status. Among working carers, self-rated health differed significantly according to the presence of additional caregivers, the use of short-stay services, and caregiver burden. Caregivers without additional support and those with a higher caregiver burden were more likely to report poor health. Among non-working carers, poorer self-rated health was observed among those with a higher caregiver burden.

### 3.5. Association Between Employment Status and Stress over the Past Month and Related Factors

Table 6 presents the association between employment status and stress over the past month after applying IPW. Employment status was significantly associated with stress over the past month (OR = 1.76, 95% CI: 1.18–2.62, *p* = 0.006), and this association remained significant after adjusting for confounding factors (OR = 3.86, 95% CI: 2.23–6.70, *p* < 0.001).

Table 7 presents the results of the exploratory univariate analyses of stress over the past month by employment status. Among working carers, stress differed significantly according to the presence of additional caregivers, with a higher proportion of stress observed among those without caregiving support. Among non-working carers, stress differed significantly according to caregiver gender, the presence of cognitive impairment in the care recipient, and the use of short-stay services. A higher proportion of stress was observed among female caregivers, those caring for individuals with cognitive impairment, and those using short-stay services.

## 4. Discussion

This study analyzed 409 family caregivers of patients receiving home-visit nursing services and examined the associations between employment status and health as well as exploratory associations between caregiving-related factors and health outcomes according to employment status. Of the study participants, 126 (30.8%) were classified as working carers. Previous studies in Japan targeting caregivers of patients receiving home-visit nursing services have reported that 20–30% of the caregivers are working carers [45,46], and the findings of the present study are consistent with those reported previously.

Using propensity score weighting, we examined the association between employment and health statuses. Univariate logistic regression analysis showed that working carers had significantly higher odds of reporting good self-rated health than non-working carers; however, this association was no longer significant after adjustment for caregiving-related factors in the multivariable analysis. A previous study on working carers of individuals with dementia reported that a greater caregiving burden, measured by Activities of Daily Living and Instrumental Activities of Daily Living, was associated with poorer self-rated health among caregivers [36]. In the present study, although we adjusted for the presence of a high level of care need (care level, ≥3), which may reflect caregiving burden, it is possible that we did not fully account for the subjective burden experienced by caregivers in their daily lives.

In the exploratory univariate analyses, higher total NAC scores were observed among caregivers with poor self-rated health in both working and non-working carers. Specifically, caregivers in the unhealthy group had higher mean total NAC score than those in the healthy group. These findings suggest that caregivers who perceive their caregiving experience more negatively are more likely to report poorer self-rated health, regardless of their employment status. Similar findings have been reported in studies of caregivers of patients with cancer, where a greater caregiver burden was associated with poorer self-rated health [15]. It should be noted that the total NAC score used in this study differs conceptually from conventional caregiver burden scales, because it captures negative perceptions of caregiving, such as distress and difficulty. Nevertheless, the findings of the present study suggest that a negative appraisal of caregiving may be an important factor associated with caregivers’ self-rated health. In other words, focusing on how caregivers perceive and assign meaning to their caregiving experiences may be important for developing effective support strategies.

In the exploratory univariate analyses among working carers, self-rated health differed according to the presence of additional caregivers and the use of short-stay services. Poor self-rated health was more common among caregivers without support from others, suggesting a link between insufficient support and poorer health. In Japan, changes in family and social structures have altered the availability of informal support from family members and others. National data have shown that the proportion of households with care recipients living in three-generation households has declined, whereas the proportions living in single-person households, couple-only households, and nuclear-family households have increased [47]. In addition, Japan’s total fertility rate declined to 1.14 in 2025, and the continuing decline in birth rates may reduce the number of family members available to share caregiving responsibilities [48]. Furthermore, employment- and commuting-based lifestyles may limit the time family members can devote to providing day-to-day care. Under these circumstances, working carers who do not have caregiving support may need to manage both employment and caregiving responsibilities, which may be associated with poorer self-rated health. Similarly, previous studies have reported that insufficient family support is associated with poorer self-rated health among working carers [29], and the findings of the present study are consistent with these findings.

When stress over the past month was examined as an outcome, univariate logistic regression analysis showed that working carers had significantly higher odds of experiencing stress than non-working carers. This association remained significant after adjusting for caregiving-related factors and the odds ratio increased after adjustment. These findings suggest that employment status was associated with stress even after adjustment for caregiving-related factors.

This result can be interpreted in light of the concept of “role strain,” proposed by William J. Goode [16]. Role strain refers to the condition in which individuals experience difficulty in fulfilling the expectations and demands of multiple social roles simultaneously. Working carers are required to fulfill both employment and caregiving roles, which may be associated with role conflict and greater stress. Although role conflict was not directly measured in this study, the higher levels of stress observed among working carers may reflect the presence of such role strain. Previous studies among carers of individuals with dementia have also reported that greater difficulty in balancing work and caregiving is associated with higher levels of caregiver role strain [25].

Furthermore, in the exploratory univariate analyses among working carers, stress differed according to the presence of additional caregivers, with a higher proportion of stress observed among those without caregiving support. Previous studies have also shown that a lack of family support is associated with increased depressive symptoms among working carers, reflecting the psychological strain of insufficient support [39]. In contrast, in exploratory univariate analyses of non-working carers, stress differed according to caregiver gender, the presence of cognitive impairment in the care recipient, and the use of short-stay services. In particular, female caregivers were more likely to report stress [29], which may reflect the concentration of caregiving responsibilities and the associated psychological burden.

This study had some limitations. First, employment status was assessed using a dichotomous variable that did not allow us to distinguish between individuals who left employment because of caregiving and those who were unable to work despite wishing to do so. As a result, the non-working carer group may include individuals whose health has deteriorated or whose caregiving burden has increased to the extent that continuing employment has become difficult. Future studies should consider more detailed aspects of employment status, such as the duration of employment, timing of interruption, and reasons for leaving work.

Second, detailed information on the work environment was limited. Employment status in this study was classified simply as working or non-working, and factors such as employment type (e.g., full-time or part-time), working hours, caregiving-to-work conflict, reduced work performance due to caregiving, and perceived difficulty in balancing work and caregiving were not fully assessed. In addition, the work environment was evaluated using limited self-reported information, and objective data on working conditions were not available. Future studies should incorporate more detailed and objective assessments of the work environment.

Third, this study used self-rated health and stress over the past month as health indicators. These measures are subjective and were based on self-reported questionnaires; no objective health indicators were included. Although subjective measures are important for understanding caregivers’ health, they may be influenced by respondents’ perceptions and psychological states. Future studies should incorporate objective measures, such as physiological indicators, to provide a more comprehensive assessment of caregiver health.

Fourth, support situations and unmet needs were not assessed using validated measures. In addition, information on socioeconomic factors, including household income and health insurance status, was not collected. These unmeasured factors may have influenced participants’ perceptions of available support and their self-reported health. Future studies should consider validated measures of support and unmet needs as well as relevant socioeconomic factors.

Finally, because this study employed a cross-sectional design, causal relationships among employment status, caregiving conditions, and health could not be established. Longitudinal studies are required to examine the influence of changes in employment and caregiving conditions on health over time.

## 5. Conclusions

This study aimed to examine the association between employment status and health and to explore associations between caregiving-related factors and health outcomes according to employment status among family caregivers of patients receiving home-visit nursing services.

Using IPW and logistic regression analysis adjusted for caregiving conditions, no significant association was found between employment status and self-rated health. In contrast, a significant positive association was observed between employment status and stress over the past month.

Exploratory stratified analyses suggested differences in self-rated health according to the presence of additional caregivers, use of short-stay services, and negative appraisal of caregiving among working carers. Among non-working carers, poorer self-rated health was observed among those with a higher negative appraisal of caregiving. Additionally, a higher proportion of stress was observed among working carers without additional caregivers. Among non-working carers, stress differed according to caregiver gender, the presence of cognitive impairment in the care recipient, and the use of short-stay services.

Overall, working carers reported higher levels of stress than those for non-working carers, even after adjustment for caregiving-related factors. In particular, caregiving support may play an important role in the health of working carers. These findings may contribute to the development of support strategies tailored to caregivers’ employment status.

## Figures and Tables

**Table 1:** Overall Characteristics of the Participants.

		Overall (*n* = 409)
	*n*	Mean ± SD Median [IQR], or *n* (%)
Caregiver Characteristics		
Caregiver’s age (years)	409	68.4 ± 11.8
<50		25 (6.1%)
50–64		123 (30.1%)
65–74		123 (30.1%)
≥75		138 (33.7%)
Caregiver’s gender	409	
Male		111 (27.1%)
Female		298 (72.9%)
Relationship with the care recipient	400	
Spouse		206 (51.5%)
Non-spouse		194 (48.5%)
Cohabiting family members	397	
Yes		294 (74.1%)
No		103 (25.9%)
Caregiving Situations		
Care recipient age	403	80.1 ± 14.6
Care recipient gender	397	
Male		189 (47.6%)
Female		208 (52.4%)
Level of care need	361	
support level 1 to care level 2		140 (38.8%)
care level 3 or higher		221 (61.2%)
Duration of caregiving (months)	408	39.0 [17.0–85.0]
<24 months		115 (28.2%)
≥24 months		293 (71.8%)
Caregiver Health		
Self-rated health	405	
Healthy		281 (69.4%)
Unhealthy		124 (30.6%)
Stress over the past month	405	
Stressed		327 (80.7%)
Not stressed		78 (19.3%)

SD, standard deviation; IQR, interquartile range. Numbers may not sum to the total because of missing or invalid responses.

**Table 2:** Characteristics of the working and non-working carers.

	Working Carer Group	Non-Working Carer Group	
	(*n* = 126)	(*n* = 283)	*p*
	Mean ± SD Median [IQR], or *n* (%)	Mean ± SD Median [IQR], or *n* (%)	
Caregiver Characteristics			
Caregiver’s age (years)	60.9 ± 10.6	71.8 ± 10.8	<0.001
<50	18 (14.3%)	7 (2.5%)	<0.001
50–64	65 (51.6%)	58 (20.5%)	
65–74	27 (21.4%)	96 (33.9%)	
≥75	16 (12.7%)	122 (43.1%)	
Caregiver’s gender			
Male	34 (27.0%)	77 (27.2%)	0.962
Female	92 (73.0%)	206 (72.8%)	
Relationship with the care recipient			
Spouse	36 (29.3%)	170 (61.4%)	<0.001
Non-spouse	87 (70.7%)	107 (38.6%)	
Cohabiting family members			
Yes	96 (78.0%)	198 (72.3%)	0.224
No	27 (22.0%)	76 (27.7%)	
Caregiving Situations			
Care recipient age	77.6 ± 17.4	81.2 ± 13.0	0.023
Care recipient gender			
Male	47 (39.5%)	142 (51.1%)	0.034
Female	72 (60.5%)	136 (48.9%)	
Level of care need			
support level 1 to care level 2	50 (49.0%)	90 (34.7%)	0.012
care level 3 or higher	52 (51.0%)	169 (65.3%)	
Duration of caregiving (months)	36.0 [12.0–85.0]	39.0 [18.0–85.0]	0.736
<24 months	35 (28.0%)	80 (28.3%)	0.956
≥24 months	90 (72.0%)	203 (71.7%)	
Caregiver Health			
Self-rated health			
Healthy	99 (79.2%)	182 (65.0%)	0.004
Unhealthy	26 (20.8%)	98 (35.0%)	
Stress over the past month			
Stressed	109 (88.6%)	218 (77.3%)	0.008
Not stressed	14 (11.4%)	64 (22.7%)	

*p* values were calculated using the chi-square test, independent samples *t*-test, or Mann–Whitney *U* test. SD, standard deviation; IQR, interquartile range.

**Table 3:** Covariate balance before and after inverse probability weighting (IPW).

	Before IPW			After IPW		
	Working Carers	Non-Working Carers	SMD	Working Carers	Non-Working Carers	SMD
	Mean ± SD	Mean ± SD		Mean ± SD	Mean ± SD	
Caregiver Characteristics						
Caregiver’s age (years)	60.88 ± 10.58	71.76 ± 10.77	−1.02	66.98 ± 10.77	66.99 ± 13.27	0.00
Caregiver’s gender						
Male (1)	0.27 ± 0.45	0.27 ± 0.45	0.00	0.30 ± 0.46	0.24 ± 0.43	0.13
Female (0)						
Relationship with the care recipient						
Spouse (1)	0.29 ± 0.46	0.61 ± 0.49	−0.68	0.48 ± 0.50	0.49 ± 0.50	−0.02
Non-spouse (0)						
Cohabiting family members						
Yes (1)	0.78 ± 0.42	0.72 ± 0.45	0.14	0.75 ± 0.43	0.75 ± 0.43	0.00
No (0)						

Values in parentheses (1 and 0) indicate the variable coding used in the statistical analyses. SD, standard deviation; SMD, standardized mean difference.

**Table 4:** Association between employment status and self-rated health after applying IPW.

	Univariate Analysis			Multivariable Analysis		
	Odds Ratio	95% Confidence Interval	*p*	Adjusted Odds Ratio	95% Confidence Interval	*p*
Employment status	1.90	1.37–2.63	<0.001	1.41	0.98–2.03	0.066

Logistic regression analysis. Independent variable: employment status (0 = non-working carers, 1 = working carers). Dependent variable: self-rated health (0 = unhealthy, 1 = healthy). Covariates: care recipient age, high care need level (care level ≥ 3), and duration of caregiving (months). Number of observations included in the univariate model: *n* = 384; multivariable model: *n* = 342.

**Table 5:** Exploratory univariate analyses of self-rated health by employment status.

		Working Carers (*n* = 126)			Non-Working Carers (*n* = 283)		
		Healthy	Unhealthy		Healthy	Unhealthy	
		*n* (%)/*n* (M ± SD)	*n* (%)/*n* (M ± SD)	*p*	*n* (%)/*n* (M ± SD)	*n* (%)/*n* (M ± SD)	*p*
Caregiver Characteristics							
	Caregiver’s age (years)	99 (60.8 ± 10.7)	26 (61.6 ± 10.2)	0.730	181 (70.9 ± 11.1)	98 (73.4 ± 10.1)	0.062
	Relationship with the care recipient						
	Spouse	29 (30.2%)	7 (26.9%)	0.745	107 (60.1%)	61 (63.5%)	0.578
	Non-spouse	67 (69.8%)	19 (73.1%)		71 (39.9%)	35 (36.5%)	
Work Environment							
	Adjustment of working hours for caregiving						
	Able to adjust	37 (38.9%)	11 (42.3%)	0.756			
	Unable to adjust	58 (61.1%)	15 (57.7%)				
Caregiving Situations							
	Daily caregiving time						
	Half-day to full-day	35 (36.1%)	12 (50.0%)	0.210	83 (47.7%)	57 (58.8%)	0.081
	≤2–3 h	62 (63.9%)	12 (50.0%)		91 (52.3%)	40 (41.2%)	
	Cognitive impairment in the care recipient						
	Present	52 (52.5%)	15 (60.0%)	0.503	102 (57.3%)	54 (57.4%)	0.982
	Absent	47 (47.5%)	10 (40.0%)		76 (42.7%)	40 (42.6%)	
Support Situations							
	Additional caregivers						
	Yes	63 (64.9%)	9 (36.0%)	0.009	74 (41.3%)	35 (35.7%)	0.359
	No	34 (35.1%)	16 (64.0%)		105 (58.7%)	63 (64.3%)	
	Use of home-based services						
	Yes	79 (79.8%)	20 (76.9%)	0.748	154 (85.1%)	83 (85.6%)	0.914
	No	20 (20.2%)	6 (23.1%)		27 (14.9%)	14 (14.4%)	
	Use of daycare services						
	Yes	39 (39.8%)	14 (56.0%)	0.144	77 (42.8%)	43 (44.8%)	0.614
	No	59 (60.2%)	11 (44.0%)		103 (57.2%)	53 (55.2%)	
	Use of short-stay services						
	Yes	15 (25.2%)	9 (34.6%)	0.046	33 (18.1%)	20 (20.6%)	0.748
	No	84 (74.8%)	17 (65.4%)		149 (81.9%)	77 (79.4%)	
Caregiver Burden							
	Negative Appraisal of Care	88 (31.2 ± 18.6)	22 (45.8 ± 19.4)	0.001	161 (33.8 ± 17.4)	79 (43.2 ± 21.1)	<0.001

*p* values were calculated using the chi-square test, independent samples *t*-test, or Fisher’s exact test. M, mean; SD, standard deviation.

**Table 6:** Association between employment status and stress over the past month after applying IPW.

	Univariate Analysis			Multivariable Analysis		
	Odds Ratio	95% Confidence Interval	*p*	Adjusted Odds Ratio	95% Confidence Interval	*p*
Employment status	1.76	1.18–2.62	0.006	3.86	2.23–6.70	<0.001

Logistic regression analysis. Independent variable: employment status (0 = non-working carers, 1 = working carers). Dependent variable: stress over the past month (0 = not stressed, 1 = stressed). Covariates: care recipient age, high care need level (care level ≥ 3), and duration of caregiving (months). Number of observations included in the univariate model: *n* = 385; multivariable model: *n* = 342.

**Table 7:** Exploratory univariate analyses of stress over the past month by employment status.

		Working Carers (*n* = 126)			Non-Working Carers (*n* = 283)		
		Stressed	Not Stressed		Stressed	Not Stressed	
		*n* (%)/*n* (M ± SD)	*n* (%)/*n* (M ± SD)	*p*	*n* (%)/*n* (M ± SD)	*n* (%)/*n* (M ± SD)	*p*
Caregiver Characteristics							
	Caregiver’s age (years)	109 (60.2 ± 10.4)	14 (63.5 ± 10.5)	0.260	217 (71.4 ± 10.8)	64 (73.3 ± 10.5)	0.231
	Caregiver’s gender						
	Male	27 (24.8%)	5 (35.7%)	0.517	50 (22.9%)	27 (42.2%)	0.002
	Female	82 (75.2%)	9 (64.3%)		168 (77.1%)	37 (57.8%)	
	Relationship with the care recipient						
	Spouse	33 (30.8%)	1 (7.7%)	0.107	128 (59.5%)	42 (67.7%)	0.242
	Non-spouse	74 (69.2%)	12 (92.3%)		87 (40.5%)	20 (32.3%)	
	Cohabiting family members						
	Yes	82 (77.4%)	11 (78.6%)	1.000	154 (72.3%)	43 (71.7%)	0.923
	No	24 (22.6%)	3 (21.4%)		59 (27.7%)	17 (28.3%)	
Work Environment							
	Adjustment of working hours for caregiving						
	Able to adjust	44 (41.5%)	4 (28.6%)	0.353			
	Unable to adjust	62 (58.5%)	10 (71.4%)				
Caregiving Situations							
	Daily caregiving time						
	Half-day to full-day	43 (40.2%)	4 (33.3%)	0.762	114 (54.5%)	26 (41.3%)	0.065
	≤2–3 h	64 (59.8%)	8 (66.7%)		95 (45.5%)	37 (58.7%)	
	Duration of caregiving (months)						
	<24 months	30 (27.8%)	3 (21.4%)	0.757	62 (28.4%)	18 (28.1%)	0.961
	≥24 months	78 (72.2%)	11 (78.6%)		156 (71.6%)	46 (71.9%)	
	Cognitive impairment in the care recipient						
	Present	60 (55.6%)	6 (42.9%)	0.107	129 (60.6%)	27 (44.3%)	0.023
	Absent	48 (44.4%)	8 (57.1%)		84 (39.4%)	34 (55.7%)	
Support Situations							
	Additional caregivers						
	Yes	59 (55.1%)	11 (84.6%)	0.042	89 (41.7%)	20 (31.7%)	0.176
	No	48 (44.9%)	2 (15.4%)		127 (58.8%)	43 (68.3%)	
	Use of home-based services						
	Yes	87 (79.8%)	10 (71.4%)	0.492	190 (87.2%)	50 (80.6%)	0.196
	No	22 (20.2%)	4 (28.6%)		28 (12.8%)	12 (19.4%)	
	Use of daycare services						
	Yes	47 (43.9%)	6 (42.9%)	0.940	95 (44.0%)	25 (40.3%)	0.608
	No	60 (56.1%)	8 (57.1%)		121 (56.0%)	37 (59.7%)	
	Use of short-stay services						
	Yes	21 (19.3%)	3 (21.4%)	0.848	47 (21.6%)	6 (9.5%)	0.031
	No	88 (80.7%)	11 (78.6%)		171 (78.4%)	57 (90.5%)	

*p* values were calculated using the chi-square test, independent samples *t*-test, or Fisher’s exact test.

## Data Availability

The data that support the findings of this study are available from the corresponding author upon reasonable request. The data are not publicly available due to privacy or ethical restrictions.
